# The potential advantages and workflow challenges of long axial field of view PET/CT

**DOI:** 10.1002/jmrs.686

**Published:** 2023-05-08

**Authors:** Wei‐Ting J. Chen, William I. D. Rae, Peter L. Kench, Steven R. Meikle

**Affiliations:** ^1^ Discipline of Medical Imaging Science, Faculty of Medicine and Health University of Sydney Camperdown New South Wales Australia; ^2^ Medical Image Optimisation and Perception Group (MIOPeG), Faculty of Medicine and Health University of Sydney Camperdown New South Wales Australia; ^3^ Medical Imaging Department Prince of Wales Hospital Randwick New South Wales Australia; ^4^ Brain and Mind Centre University of Sydney Camperdown New South Wales Australia

## Abstract

Recently developed Long (≥100 cm) axial field of view (AFOV) PET/CT scanners are capable of producing images with higher signal‐to‐noise ratio, or performing faster whole‐body acquisitions, or scanning with lower radiation dose to the patient, compared with conventional PET/CT scanners. These benefits, which arise due to their substantially higher, by more than an order of magnitude, geometric efficiency, have been well described in the recent literature. The introduction of Long AFOV PET/CT technology into the clinic also has important implications for the design and workflow of PET/CT facilities and their effects on radiation exposure to staff and patients. Maximising the considerable benefits of this technology requires a thorough understanding of the relationships between these factors to optimise workflows while appropriately managing radiation exposure. This article reviews current knowledge on PET/CT facility design, workflows and their effects on radiation exposure, identifies gaps in the literature and discusses the challenges that need to be considered with the introduction of Long AFOV PET/CT into the clinic.

## Graphical abstract

Recently developed Long (≥100 cm) axial field of view (AFOV) PET/CT scanners are capable of faster whole‐body acquisitions or scanning with lower radiation dose to the patient due to their substantially higher geometric efficiency. The introduction of Long AFOV PET/CT technology into the clinic also has important implications for the design and workflow of PET/CT facilities and their effects on radiation exposure to staff and patients. This article reviews current knowledge on PET/CT facility design, workflows, and their effects on radiation exposure, identifies gaps in the literature and discusses the challenges that need to be considered with the introduction of Long AFOV PET/CT into the clinic.

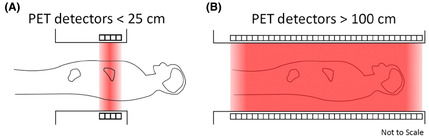

## Introduction

**Figure 1:**
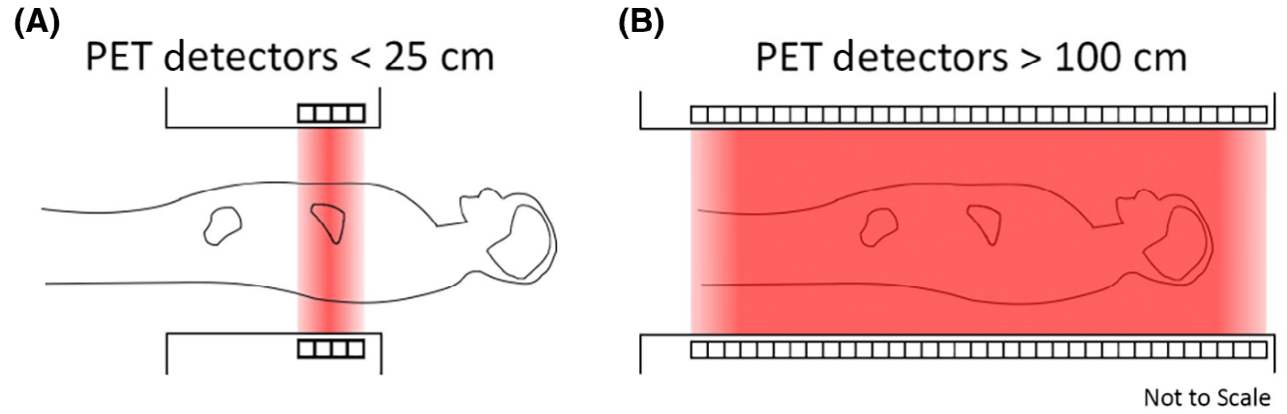
Compared with the limited sensitivity of (A) current generation PET/CT, the (B) Long AFOV PET/CT dramatically increases the system sensitivity for imaging the entire body.

Positron emission tomography/computed tomography (PET/CT) provides physiological and functional information about the body using a non‐invasive imaging technique based on the localisation of trace amounts of a positron‐emitting radiopharmaceutical administered to the patient.^1^ However, current PET/CT scanners are restricted by limited signal‐to‐noise ratio (SNR), relatively high radiation dose and long scanning times compared to other imaging modalities.^2^ This is especially evident when performing PET/CT scans of the whole body, which is a routine cancer staging and treatment response evaluation procedure.^3^ The axial field of view (AFOV) of a typical PET scanner is approximately 15–25 cm which limits the signal it can capture at one time to that emanating from a corresponding 15–25 cm thick section of the body. This results in inefficient detection of the administered radioactivity when imaging from head to thigh which is a common protocol in clinical practice.^2^ Recent technological advancements in the development of Long AFOV PET/CT scanners, also known as Total‐Body PET/CT scanners, have led to systems with markedly increased AFOV; greater than or equal to 100 cm. This increased AFOV results in a substantially increased geometric efficiency for detecting the annihilation radiation emanating from the patient. For example, the uExplorer (United Imaging Healthcare, Shanghai, China) has an AFOV of 1.94 m.^4^ This allows the entire adult human body to be scanned in a single bed position (Fig. 1). Similarly, the PennPET Explorer is planned to have an AFOV of 1.4 m,^5^ and the Siemens Biograph Vision Quadra has an AFOV of 1.06 m (Siemens Healthineers, Knoxville, USA).^6^

Compared to current generation PET/CT scanners, the sensitivity of such scanners is greater by more than an order of magnitude, as reported in NEMA performance studies^7^, ^8^ and confirmed in initial head‐to‐head clinical comparisons.^6^ The improved system sensitivity of the Long AFOV PET/CT scanner allows greater flexibility to optimise the image acquisition in terms of image quality, radiation exposure to the patient and acquisition time, all of which are interrelated.^2^ For example, the imaging team may reduce the radiation dose to the patient while maintaining the same acquisition time and image quality as that of current PET/CT scanners.^2^ Decreasing the amount of injected activity may also reduce radiopharmaceutical costs. Alternatively, instead of opting for dose reduction, the imaging team may decide to administer the same radiopharmaceutical activity as for current PET/CT protocols to reduce the acquisition time, thereby limiting both voluntary and involuntary movement that may introduce artefacts in the images for paediatric patients and uncooperative patients. Another option is to use the same acquisition time and administered dose as current protocols and acquire a higher quality image for diagnostic purposes or even alter some or all parameters to customise the protocol for each patient. In practice, it is anticipated that some balance between these competing parameters will be achieved, and the choice of appropriate balance will likely be different for different sites.

The optimisation of multiple acquisition parameters is challenging. Yet, such changes can lead to an overall improvement in the workflow of a PET/CT department in terms of reduced patient dose, reduced staff dose, shorter acquisition times, optimised image quality, reduced radiopharmaceutical costs, as well as increased patient throughput.^9^ However, the optimal balance of these benefits will still have to be determined using a considered, iterative process involving all imaging team members. Simultaneously, new challenges will arise for patient management in the department to maintain safe radiation practice without compromising patient outcomes. The currently limited literature on Long AFOV PET/CT scanners relates mainly to reports of scanner performance and the first human studies. Whilst there is some literature on optimising PET department workflows using current generation PET/CT scanners,^10^, ^11^ there are no such studies for Long AFOV PET/CT.

This article reviews the recent literature on the optimisation of various components of PET/CT clinical workflow while identifying the potential workflow optimisation challenges that may arise when transitioning from current generation PET/CT systems to Long AFOV PET/CT technology. The anticipated improvement in image quality and, thus, the possibility of identifying previously unseen micro‐metastases and anatomical variants may also create challenges in terms of clinical assessment of the images. The clinical significance of such additional information will need to be evaluated over time. This improvement will also impact the optimisation of the imaging process, including acquisition and reconstruction parameters, as well as the administered activity. However, this article will not address these challenges and will instead focus on the workflow aspects of Long AFOV PET/CT.

## PET/CT workflows

**Figure 2:**
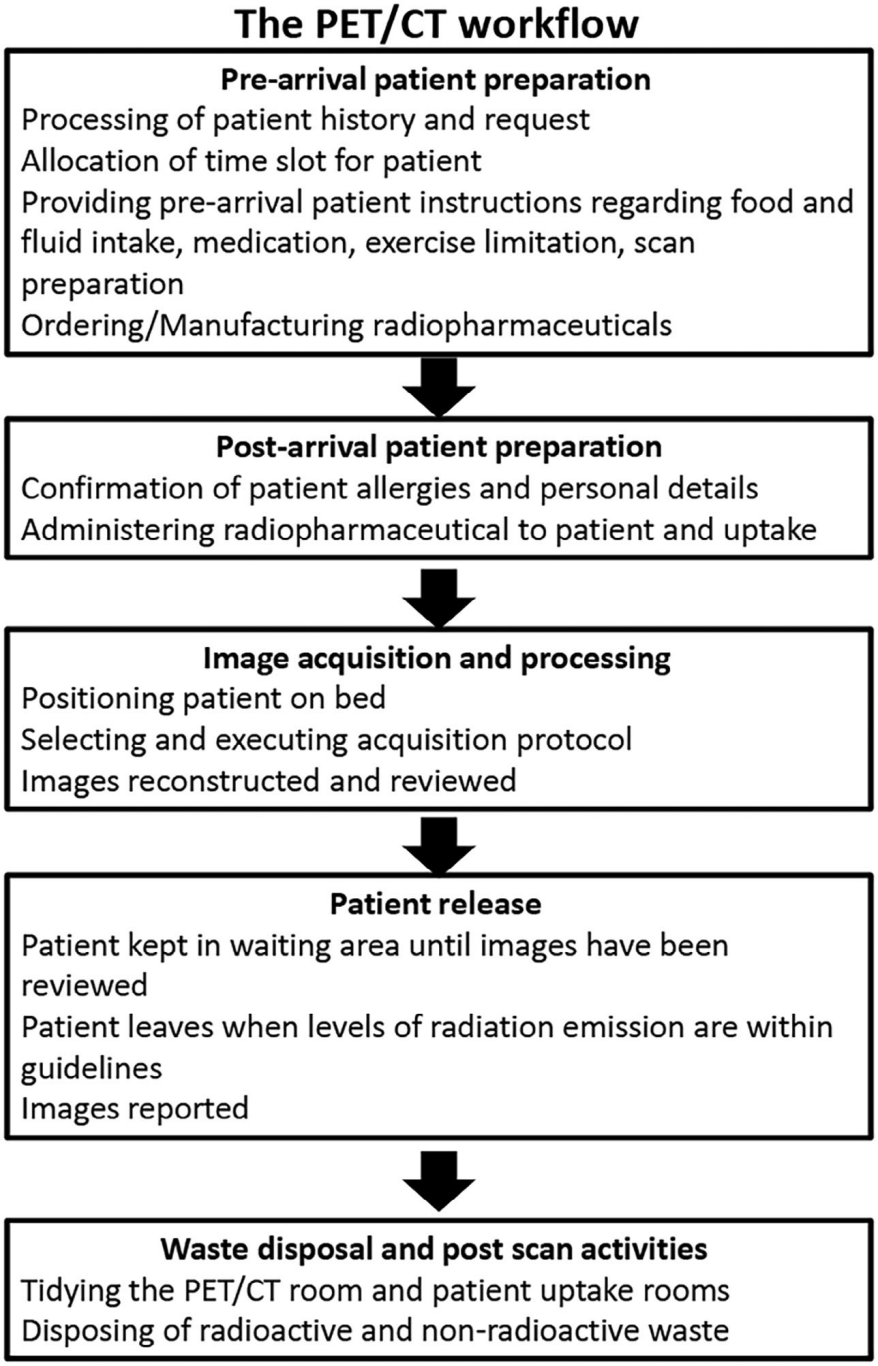
Summary of the workflow steps and considerations at a generic PET/CT facility.

There are five main workflow stages for each PET/CT scan (Fig. 2):

1. Pre‐arrival patient preparation
2. Post‐arrival patient preparation
3. Image acquisition and processing
4. Patient release
5. Waste disposal and post scan activities

In the pre‐arrival patient preparation stage, a camera time slot is allocated for each patient and the patient is given pre‐scan instructions, such as fasting overnight as appropriate. A radiopharmaceutical is also ordered or produced in‐house for the scan. Upon arrival in the department, the patient is injected with the radiopharmaceutical and waits in a shielded uptake room for up to 1 hour. For certain studies such as prostate‐specific membrane antigen (PSMA) imaging, the patient may wait in the room for over an hour depending on departmental protocols.

The patient is then taken to the PET/CT scanner for image acquisition while the imaging team chooses the most appropriate imaging protocol. For dynamic scans, the patient is injected with the radiopharmaceutical in the PET/CT camera room. After the scan, the patient is taken to a waiting area and leaves after the imaging staff have reviewed the images. Following PET imaging and review, the patient's radioactivity level is usually sufficiently low for the patient to leave immediately. The standard processes after the patient leaves are carried out, such as changing bed coverings, disposal of any waste which may or may not be radioactive, and any administrative procedures which are typically required.

With the introduction of Long AFOV PET/CT, various aspects of the protocol need to be optimised to enhance the workflow of the department and the diagnostic value of the scans. For example, the uptake time is one parameter that may need to be optimised. Current uptake times for FDG scanning are a trade‐off between its slow uptake in cancer cells, clearance from background and the sensitivity of current PET systems which limit the uptake time to about 1‐hour post‐injection due to noise constraints. With the substantially increased sensitivity of Long AFOV PET/CT, extending uptake times to over an hour for FDG patients is possible and can be expected to improve the contrast of lesions.^12^ However, such a change would require additional uptake rooms to maintain the same patient throughput as current PET/CT systems. Furthermore, parameters that were previously constant can be customised for each patient. Acquisition times may be reduced to limit motion artefacts while increasing patient throughput and the injected activity can be considerably reduced to decrease the radiation dose received by patients and staff. The radioactive waste discharged through the toilet following the administration of the activity and bladder emptying pre‐scanning will increase if the number of patients is increased. The number and detail of these activities will also impact workload and workflow and may be considerably modified by the use of Long AFOV PET/CT.

Therefore, considerable workflow changes are expected for Long AFOV PET/CT compared to current generation PET/CT in order to optimise workflow efficiency.

### PET/CT facility design

The layout of a PET/CT facility is fundamental to the workflow efficiency of the clinic. In the recent literature, there are single‐site studies highlighting the benefits of particular PET/CT facility designs.^10^, ^11^ These articles quote guidelines from governing bodies such as the American Association of Physicists in Medicine Task Group 108^13^ on which to base their facility specifications. Meanwhile, the Australian Association of Nuclear Medicine Specialists (AANMS) has published standards for accreditation of nuclear medicine sites established for Medicare reimbursement for PET services.^14^ It must be noted that the report cannot dictate site specifications since these are unique to each site. The standards are generalised and require the site to have licensed and qualified staff to develop their own site‐specific protocols.^14^ Currently, acquisition protocols are developed based on the experience of qualified staff to ensure that diagnostic PET/CT images are acquired with minimal radiation exposure. This has created a wide range of site‐specific ‘optimised’ workflow and facility designs in different clinics.^15^ This demonstrates that despite the importance of designing PET/CT facilities to optimise workflow and patient outcomes, no literature specifically evaluates these related factors. A new recommendation from the relevant expert would be especially important when designing a clinical Long AFOV PET/CT system to ensure an optimised workflow.

### Balancing acquisition and image quality optimisation

There are significant benefits of reduced acquisition time resulting in increased patient throughput and comfort when using Long AFOV PET/CT. However, patient outcomes should not be negatively impacted or injected activity unnecessarily increased. Yet, despite a greater focus on aspects of protocol optimisation in the literature, there is still insufficient information on which to base a clear optimisation strategy. The recent literature advocates using patient‐specific factors to optimise PET/CT protocols.^16^, ^17^, ^18^, ^19^ In these studies, the authors developed injected activity calculations based on patient weight and BMI. However, these studies each only examined a single scanner at a single site without demonstrating a clear relationship between acquisition time, injected activity and image quality with regard to patient size. It may be justifiable to define acquisition parameters using the longest acquisition time for paediatric patients to minimise the injected activity required and reduce the radiation dose to the patient.^17^ However, having an increased acquisition time is not without consequence. The resultant images are likely to have more artefacts caused by voluntary and involuntary motion.

It is expected that Long AFOV PET/CT may considerably reduce acquisition time to limit motion artefacts.^20^ Motion correction is sometimes used to minimise motion artefacts. The registration‐based correction method uses a spatial registration algorithm applied to reconstructed image frames to estimate and correct the movement between each frame.^21^ Meanwhile, the line‐of‐response re‐binning method enables correction for movement within each frame by manipulating the list mode coincidence events based on information from an optical motion tracker.^22^ Given recent improvements in spatial resolution in PET/CT, the requirement for accurate motion correction is becoming increasingly important and current motion correction methods may not be sufficiently effective.^20^ It is expected that Long AFOV PET/CT may considerably reduce acquisition time, therefore limiting motion artefacts.^20^

Another method for image quality optimisation is to base acquisition parameters on a pre‐defined image quality index such as Noise Equivalent Count Rates (NECR).^16^, ^23^ This method is also not without its limitations as it evaluates the count‐rate performance of a scanner which does not necessarily relate directly to diagnostic image quality. As such, a reader study is necessary to determine the diagnostic value of an image set derived using an image quality index. Meanwhile, another study found that NECR curves could be extrapolated from anthropomorphic phantoms to individual patients using object‐independent scaling factors based on patient weight to calculate an optimal injected dose for each patient.^24^ However, inferring appropriate doses for heavier patients using this method was found to be inaccurate.^24^ Therefore, a greater understanding of the patient and instrumentation factors affecting protocol optimisation is essential to streamline all aspects of the workflow of a PET/CT department.

### Radiation exposure to patients and establishing dose reference levels

The development of diagnostic reference levels (DRL) is essential for reducing the PET/CT radiation dose to patients by allowing facility comparisons to a national standard.^25^ There are published PET/CT DRLs for Australia,^26^ USA^27^, ^28^ and multiple countries in Europe^29^, ^30^ and Asia.^25^ The National DRLs published by the Australian Radiation Protection and Nuclear Safety Agency (ARPANSA) include both a PET and CT component. The DRL for the PET component is measured as the injected radioactivity measured in Becquerels while the CT component is measured either as CT dose index (CTDI) or dose length product (DLP) for whole‐body scans.^26^ The National Diagnostic Reference Levels (NDRLs) do not measure the combined effective dose received by a patient; instead, they indicate radiation exposure in the PET and CT components of the acquisition separately. As such, the NDRLs identify the need for a facility to optimise their PET/CT acquisition protocol or update equipment to prevent unnecessary radiation exposure to the patient. However, additional data on the total effective dose received by the patient would permit tailoring individual patient imaging protocols for workflow optimisation.

There are significant differences in both PET/CT acquisition protocols and imaging equipment among facilities, leading to considerable variations in the recorded dose levels at different clinics. Korea (2016), the UK (2015, 2017), Australia (2009) and New Zealand (2009) had relatively similar NDRLs for adult whole‐body ^18^F‐FDG examinations for the PET component of the scan ranging from 350 to 385 MBq while France had records of NDRLs of 250 and 260 MBq in 2012 and 2017, respectively.^25^ Meanwhile, a survey (2008–2012) of 95 PET facilities in the USA found that the recommended NDRL for whole‐body 18F‐FDG scans was 592 MBq, which is approximately 90% more than the Australian survey (2014–2015) of 19 PET facilities resulting in an NDRL of 310 MBq.^26^, ^28^, ^31^ The history of technological advancements reducing DRLs and improving image quality is well described in the literature.^20^ The introduction of Long AFOV PET/CT as the latest technological advancement is also perhaps the most consequential. The considerable increase in scanner sensitivity has extended the parameter space in such a way that DRLs may be further reduced and the quality of acquisitions improved. However, the workflow of the department will need to be altered to optimally use the extended parameter space. As such, NDRLs for Long AFOV PET/CT should also be reported separately from current generation PET/CT.

The DRLs of the CT component of the scan is even more variable than the PET component. For the CT component of current PET/CT systems, the DLP NDRLs for the UK, Korea and France are 400, 560 and 750 mGy.cm, respectively.^25^ Meanwhile, the DLP NDRL for Australia for eyes‐to‐thighs scans is 540 mGy.cm.^26^ The considerable range of NDRLs for the CT component demonstrates the potential for significant reductions in radiation dose to the patient by reducing the exposure factors during the CT scan. Therefore, dose optimisation techniques for both the PET and CT components are required to minimise the dose received by the patient.

### Radiation exposure to other allied medical workers and the general public

Examining and adjusting the radiation dose received by patients would also affect the radiation exposure of other allied medical workers and the general public passing through a PET/CT facility. ARPANSA regulates that patients should have less than 25 μSv/h measured at a distance of 1 m when leaving the facility,^32^ the European Commission Radiation Protection asserts that patients should have less than 20 μSv/h,^33^ while the United States recommends less than 50 μSv/h.^34^

The recent literature finds that the general public is exposed to minimal radiation from discharged patients. The average estimated whole‐body radiation dose rate 1 m away from a patient undergoing an ^18^F‐FDG whole‐body scan was 20.4, 21.6, 21.0, 18.0 μSv/h for the neck, chest, abdomen and proximal thigh, respectively.^35^ However, the dose rates were extrapolated from the measured external radiation dose of the patients during uptake instead of the external radiation dose post‐discharge. As such, the actual effect of PET patients on the radiation exposure to the general public remains unclear.

A consequence of the potential increased patient throughput is the challenge for PET/CT facilities to increase the number of required uptake rooms where physical space is limited. To maximise patient throughput, staff may need to keep patients injected with radiopharmaceuticals in areas with a greater population density which could lead to increased radiation exposure to other allied medical workers and the general public. There is no published English literature covering these considerations.

### Radiation exposure to PET/CT technologists

Apart from the radiation exposure to patients and the general public, there have also been recent studies on the occupational radiation dose received by staff working in current PET/CT facilities. The patient throughput, activity of radiopharmaceutical injected and the facility design were identified factors affecting the radiation dose received by staff.^36^ Technologists are exposed to radiation levels within limits set by their regulatory bodies.^36^, ^37^, ^38^ However, there are concerns about the dose received by technologists to the extremities and more radiosensitive areas, for example fingers and eyes, respectively.^39^, ^40^ Dispensing radiopharmaceuticals behind a benchtop shield offers sufficient shielding for the torso but less protection to the fingers and eyes, which may result in exceeding safe dose limits.^41^, ^42^, ^43^ The current concerns regarding staff radiation dose to extremities have to some extent been addressed by automatic radiopharmaceutical dispensers for 18F‐FDG scans.^10^, ^44^, ^45^ Since the radiation dose received by the eyes is mainly during dispensing and the injection of the radiopharmaceutical where the staff are closest to the radiopharmaceutical,^43^ using an automatic dispenser would also be expected to reduce the radiation exposure to the eyes.^46^

While some solutions exist for managing radiation risks in current PET/CT facilities new risks may arise when transitioning to a Long AFOV PET/CT system with the potential for much higher patient throughput. The effective dose to the technologist in the scanning room may be reduced since the longer AFOV would shield the technologist when the patient is inside the scanner. Yet, new radiation safety risks may result from elevated radiation exposure rates, from the increased patient throughput. When considering a reduction in the injected activity for Long AFOV PET/CT to counteract the increased dose to staff due to an anticipated increase in patient throughput, it cannot be assumed that the dose received by technologists is linearly related to injected activity. This is because of constraints such as a lack of uptake rooms or toilets which may result in staff spending more time in close contact with patients than would otherwise be the case. Since the Long AFOV PET/CT may improve patient throughput, a challenge for all PET/CT centres will be to optimise workflow without significantly increasing risks to staff and the general public. Therefore, potential radiation risks associated with an increased patient throughput due to Long AFOV PET/CT must be considered.

### Radiopharmaceutical costs

Understanding the costs associated with running a PET/CT facility is also necessary to optimise the workflow of the clinic. Radiopharmaceutical costs are dependent mainly on the amount ordered. The literature does show that performing PET acquisitions with a reduced injected activity is possible^47^ but the reduction in radiopharmaceutical costs was not assessed. A Long AFOV PET/CT facility operating with protocols requiring a lower injected activity could considerably save on radiopharmaceutical costs. As such, it would be beneficial to consider the potential cost reductions in reducing injected activity when no detriment to patient outcomes is demonstrated.

### Long AFOV PET/CT – workflow challenges

Currently, there is no literature surrounding workflow optimisation of new Long AFOV PET/CT scanners as an emerging technology. The recent literature focuses on an analysis of anticipated and actual improvements in image quality and acquisition time compared to current PET/CT systems.^2^, ^4^, ^48^ These studies do not address the potential impact on radiation exposure due to increased patient throughput or changes in facility design that improve the efficiency of a PET/CT department.

The Long AFOV PET/CT requires a well‐designed clinic to exploit the full potential of this new technology. Increased patient throughput would require more uptake rooms, toilets and staff. For example, suppose a facility requires patients to have a 1‐hour uptake time, with each scan taking 20 min, including patient positioning. In that case, three uptake rooms are required to maximise the utilisation of the scanner. If the number of uptake rooms is capped at three and the uptake time is unchanged, reducing the scanning time to less than 20 min would leave the scanner idle between patients. Under this scenario, three patients per hour is the maximum practical throughput. If the facility has six uptake rooms, the scanning time can be reduced to 10 min (including positioning and change over time), and patient throughput increases up to 6 patients per hour, assuming there are no delays in the department. Furthermore, additional physicians may also be required to increase the reporting rate to facilitate the increased patient throughput. These resources constrain the maximum patient throughput for the workflow optimisation of any PET/CT facility.

There will also be challenges regarding the Information Technology side of a PET/CT facility. To maximise the potential increase in patient throughput, the computer processing capabilities for image acquisition, reconstruction and storage must not impede the workflow of the system and become the rate‐limiting steps.

Ideally, a Long AFOV PET/CT facility would successfully optimise the balance between acquisition time, image quality and radiation dose. However, each patient's time at a PET/CT facility is not limited to the acquisition time. Technologists need to explain the procedure to the patient, position the patient on the scanner and then remove them from the scanner. Currently, this is expected to take around 3–10 min for all PET/CT systems. As such, the constraint of a practical minimum time for patient positioning and preparation to be safely conducted is anticipated when optimising workflow.

The ‘ideal’ PET/CT workflow is also subject to the interests of patients and the operator of the PET/CT facility. Current guidelines for medical radiation safety are based on the ALARA principle where minimising the injected activity is driven by risk versus benefit considerations for the patient. However, the capital cost of a Long AFOV PET/CT is up to US$10 million compared to US$1.5–2 million for a current generation PET/CT scanner.^2^ The higher upfront capital and ongoing maintenance costs of a Long AFOV PET/CT scanner may need to be recovered by increasing patient throughput and lowering consumable (radiopharmaceutical) costs. Increasing patient throughput may also impose additional costs in increased staffing to ensure safety and comfort for the patients.

The Long AFOV PET/CT technological advancements will undoubtedly impact protocols, patient throughput and image quality. There are many options to consider when designing new, optimised workflows. A single optimisation strategy is unlikely to apply uniformly across all facilities due to considerable variations in caseload, regulations and reimbursement policies across jurisdictions. However, a clear understanding of the factors affecting workflow will enable local optimisation for best practice in patient care and the safety of staff and the general public.

### Limitations

The optimisation of Long AFOV PET/CT systems for clinical use is still at the early stages. As such, an informed assessment of how Long AFOV PET/CT systems are likely to impact workflows is difficult to undertake at this stage, although this review attempts to highlight the key issues. In addition, the interdependencies of acquisition parameters and the many anticipated workflow considerations are considerable for Long AFOV PET/CT (i.e. the parameter space is large). One approach to this problem may be to focus on the protocols of existing LAFOV PET sites (i.e. separately from greenfield sites) and reducing the parameter space by combining injected activity and acquisition time into a single parameter (dose * scan_time). This would enable one to study the effect of a given protocol on local workflow and analyse this relationship across sites. However, one also needs to factor in performance differences between different LAFOV systems which may confound the effects of acquisition parameters on workflow optimisation. Indeed, there remain gaps in the literature on workflow optimisation even for current generation PET/CT technology. Furthermore, there are relatively few Long AFOV PET/CT systems in current operation. Within these limitations, this review aimed to highlight the challenges that must be addressed to optimise workflow and address the challenges associated with the implementation of Long AFOV PET/CT in a clinical environment.

## Conclusion

Although many studies have reported on the optimisation of current PET/CT systems in relation to acquisition and reconstruction parameters, there are relatively few studies on the impact of these factors on the workflow efficiency of a PET/CT department. Furthermore, more recent studies on Long AFOV PET/CT technology do not address the potential impact on workflow, with its potential for much higher patient throughput and/or lower radiation exposure. Based on these gaps identified in the literature, further research aimed at investigating the effects of the introduction of Long AFOV PET/CT in terms of the radiation dose to the staff and patient as well as the impacts on the workflow of the department is needed for clinically optimised transition to Long AFOV PET/CT.

## Conflicts of Interest

The authors certify that they have no affiliations with or involvement in any organisation or entity with any financial interests or non‐financial interests in the subject matter or materials discussed in this article.
